# Cycloid Psychosis: Differential Diagnosis and case repot

**DOI:** 10.1192/j.eurpsy.2026.11795

**Published:** 2026-07-31

**Authors:** P. Nava Garcia, M. Arevalo Gil, S. Orrego Molina, F. Pascual Pinazo, M. Sobredo Vega

**Affiliations:** Psychiatry, Hospital Universitario Infanta Leonor, Madrid, Spain

## Abstract

**Introduction:**

A **43-year-old woman** is admitted to the Hospitalization Unit due to a **psychotic episode** characterized by **marked behavioral disorganization**, psychomotor agitation, irritability, suspiciousness, pressuring, verbose, rambling speech with loose associations, from which **megalomaniacal and paranoid delusional content** emerges. Global insomnia with a significant increase in anxiety. A **family stressor** is identified as a precipitant.

Two years prior, the patient presented with a **similar episode** requiring brief hospital admission, stemming from high work stress. She was treated with **Olanzapine 10mg** with a rapid response within the first 24-48 hours and complete symptomatic resolution (*ad integrum*). She discontinued neuroleptic treatment after one week and has maintained stability until now.

**Objectives:**

To establish a differential diagnosis and according treatment.

**Methods:**

There are no relevant medical history records. No toxic substance consumption. Laboratory and imaging tests are within normal limits.

A first-degree relative in the horizontal line has a **Bipolar Disorder** diagnosis, with sustained stability described without treatment.

**Results:**

The first episode was classified as a **psychogenic disorder**. The current one initially appears as a **maniform decompensation** with psychotic symptoms: congruent and incongruent delusions, and hallucinatory phenomena. The response to antipsychotic treatment with **Olanzapine 10mg** is rapid (first 24-48h) and complete, although it is substituted with **Aripiprazole** due to excessive somnolence. Upon hospital discharge, the treatment is **Aripiprazole 300mg IM** (Intramuscular).

In this case, for which other diagnostic hypotheses were initially proposed, the **differential diagnosis with Cycloid Psychosis** is consideredTo e

**Conclusions:**

Perris and Brockington establish the following diagnostic criteria for **Cycloid Psychosis:**

- **Psychotic disorder** not related to toxic substance consumption or an organic disease.
- **Onset** between the ages of 15 and 50.
- **Sudden and rapidly progressive onset**.
- **Co-occurrence of at least 4** of the following: confusion, incongruent affect/mood, delusional ideation, sensory-perceptual disturbances, anxiety, feelings of euphoria, psychomotor alterations, death/suicidal ideation, or affective oscillations; and **frequent change in symptomatic manifestation**

**Disclosure of Interest:**

None Declared

